# Poor false sleep feedback does not affect pre-sleep cognitive arousal or subjective sleep continuity in healthy sleepers: a pilot study

**DOI:** 10.1007/s41105-022-00390-9

**Published:** 2022-05-05

**Authors:** Amelia R. Robson, Jason G. Ellis, Greg J. Elder

**Affiliations:** Northumbria Sleep Research, Department of Psychology, Faculty of Health and Life Sciences, Northumbria University, Newcastle, NE1 8ST United Kingdom

**Keywords:** Sleep feedback, Sleep tracker, Pre-sleep cognitive arousal

## Abstract

Modern wearable devices calculate a numerical metric of sleep quality (sleep feedback), which are intended to allow users to monitor and, potentially, improve their sleep. This feedback may have a negative impact on pre-sleep cognitive arousal, and subjective sleep, even in healthy sleepers, but it is not known if this is the case. This pilot study examined the impact of poor false sleep feedback, upon pre-sleep arousal and subjective sleep continuity in healthy sleepers. A total of 54 healthy sleepers (*M*_age_ = 30.19 years; SD_age_ = 12.94 years) were randomly allocated to receive good, or poor, false sleep feedback, in the form of a numerical sleep score. Participants were informed that this feedback was a true reflection of their habitual sleep. Pre-sleep cognitive and somatic arousal was measured at baseline, immediately after the presentation of the feedback, and one week afterwards. Subjective sleep continuity was measured using sleep diaries for one week before, and after, the presentation of the feedback. There were no significant differences between good and poor feedback groups in terms of pre-sleep cognitive arousal, or subjective sleep continuity, before or after the presentation of the sleep feedback. The presentation of false sleep feedback, irrespective of direction (good vs. poor) does not negatively affect pre-sleep cognitive arousal or subjective sleep continuity in healthy sleepers. Whilst the one-off presentation of sleep feedback does not negatively affect subjective sleep, the impact of more frequent sleep feedback on sleep should be examined.

## Introduction

The daily use of wearable digital devices has become increasingly common in modern society: for example, approximately 30% of American households own a wearable digital device such as a smart watch or fitness tracker [1]. Wearable devices are now increasingly used as part of the “quantified self” movement, whereby individuals regularly use wearable digital device data and quantitative metrics to measure, and enhance, their health and well-being [2].

This is the case with sleep. Many wearable devices, which are marketed to the general public, claim to be able to accurately measure sleep, and also reliably detect and distinguish between “light sleep” and “deep sleep” [3]. Many of these wearable devices also calculate a numerical metric of sleep quality (i.e. “sleep feedback”), which is derived from overnight activity data, and fed back to users [3]. For example, one commercially-available device provides users with sleep feedback in the form of automatically-calculated numerical sleep quality scores ranging from 0 to 100, where higher scores represent “better” sleep [4]. Some device manufacturers claim that if users continuously monitor their automatically-calculated device sleep feedback, they can improve their sleep by acting upon this device-led sleep feedback. [3]. This is despite the fact that most sleep metrics, which are used to provide feedback, are not validated against accurate sleep measurement techniques, and the underlying algorithms have not been validated for these purposes [3].

The monitoring of device-derived sleep feedback can potentially have a negative impact on sleep, even in healthy sleepers. This is particularly likely to be the case where users are actively encouraged to monitor, and improve, their sleep feedback [3]. Previous studies have demonstrated that selective attention towards salient sleep-related cues, such as clocks, can potentially cause and maintain the clinical sleep problem of insomnia disorder [5, 6]. Even in healthy sleepers, monitoring clocks can increase levels of pre-sleep worry, and can subsequently disturb subjective sleep [7]. Additionally, the monitoring of sleep feedback is likely to increase pre-sleep cognitive activity immediately prior to sleep, which can negatively subjective and objective sleep [8–10].

Taken together, sleep feedback, as a salient sleep-related cue, is potentially likely to influence subjective sleep, even in healthy sleepers. This is important as recent evidence demonstrates that individuals who are preoccupied with improving, or perfecting, their sleep on the basis of device-generated sleep feedback, can develop sleep problems which in the longer term may require treatment [11]. Specifically, when individuals believe there is an association between sleep feedback and their own subjective experience of sleep, or their subsequent daytime performance, this can cause “*a perfectionistic quest for the ideal sleep*” [p. 351; 11].

Although it is possible that sleep feedback can negatively affect pre-sleep cognitive arousal, or subjective sleep, to date, no studies have examined if this is the case in healthy sleepers. In one study, people with insomnia disorder who were provided with negative device-generated sleep feedback showed self-rated daytime function impairments accompanied by increased sleepiness and fatigue, relative to those who experienced positive feedback. That said, the effects on subjective sleep were not specifically examined, and no studies have examined the effect of subjectively-derived feedback [12]. Therefore, studies which examine if subjectively-derived sleep feedback can affect subjective sleep are necessary in healthy good sleepers.

The aim of the present pilot study was to examine if the presentation of poor false sleep feedback, which participants were told was a sleep metric derived from the measurement of their habitual subjective sleep (sleep diaries) could negatively affect pre-sleep arousal and subjective sleep continuity. It was hypothesised that poor sleep feedback would: (1) result in higher levels of pre-sleep cognitive arousal, and (2) negatively affect subjective sleep continuity (total sleep time and sleep efficiency), compared to good feedback.

## Materials and methods

### Participants

A total of 54 healthy sleeper participants (*M*_age_ = 30.19 years; SD_age_ = 12.94 years) were recruited from the staff and student population of Northumbria University, and from the general population using social media advertisement. This sample size was based on an a priori power analysis, conducted using G*Power 3.1 [13], which indicated that a minimum of 34 participants (*n* = 17 per group) were required to obtain an expected effect size of *f*^2^ = 0.25 (at 80% power; *α* = 0.05).

Participants were eligible to take part if they were (1) currently self-reported healthy good sleepers with a stable sleep–wake pattern; and were (2) aged 18–60 years of age. Participants were not eligible if they had (1) a self-reported history of a sleep disorder, or sleep problems, or current symptoms of these; (2) were taking sleep medication, or (3) current shift workers. All participants provided written informed consent, and the study was approved by the Northumbria University Faculty of Health and Life Sciences ethics committee. Participants were not renumerated.

### Measures

To provide an indication of habitual subjective sleep quality and subjective anxiety and depression, participants completed the Pittsburgh Sleep Quality Index [PSQI; 14], the Patient Health Questionnaire [PHQ-9; 15] and the Generalised Anxiety Disorder Questionnaire [GAD-7; 16]. To measure the usual intensity of cognitive and somatic arousal immediately prior to sleep, participants completed the Pre-Sleep Arousal Scale [PSAS; 17].

Consensus Sleep Diaries [CSD-M; 18] were used to obtain measures of subjective sleep continuity: total sleep time (TST); time in bed (TIB), sleep efficiency (SE%), which was calculated as (TST/TIB × 100); sleep onset latency (SOL); the number of awakenings (NWAK); and wake after sleep onset (WASO). Participants completed the Spielberger State-Trait Anxiety Inventory [STAI-SF; 19], where the six-item short-form version of the state scale was used to assess situational (state) anxiety symptoms, at the same time as the CSD-M.

## Procedure

**Fig. 1:**
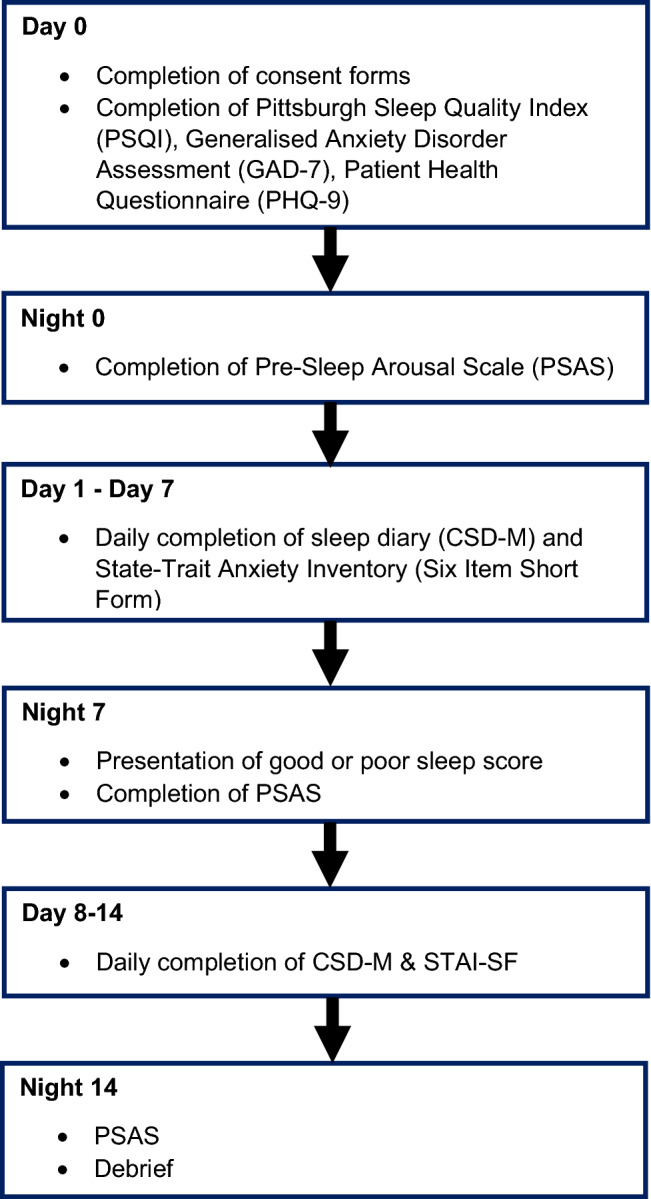
Study procedure

This study was delivered online (Qualtrics, Provo, UT) and the study procedure is summarised in Fig. 1.

On day 0, after providing informed consent, participants were randomly allocated to receive either good sleep feedback (*n* = 25), or poor sleep feedback (*n* = 24). The allocation sequence was automatically generated by Qualtrics software without influence from any member of the research team. At this stage, participants were asked to remove any sleep-tracking devices and temporarily disable any sleep-tracking phone applications that they habitually used to monitor their sleep, for the duration of the study.

Participants then completed the PSQI, PHQ-9 and GAD-7, and were asked to complete the PSAS immediately before going to sleep on Night 0. On each subsequent morning (day 1–day 7), participants completed the CSD-M and STAI-SF. On Night 7, participants were sent either good or poor fictional sleep feedback, which was automatically sent to their personal e-mail address, then repeated the PSAS. Participants completed the CSD-M and STAI-SF from day 8–14. On Night 14, participants repeated the PSAS and received a full debrief by e-mail.

### Sleep feedback

**Fig. 2:**
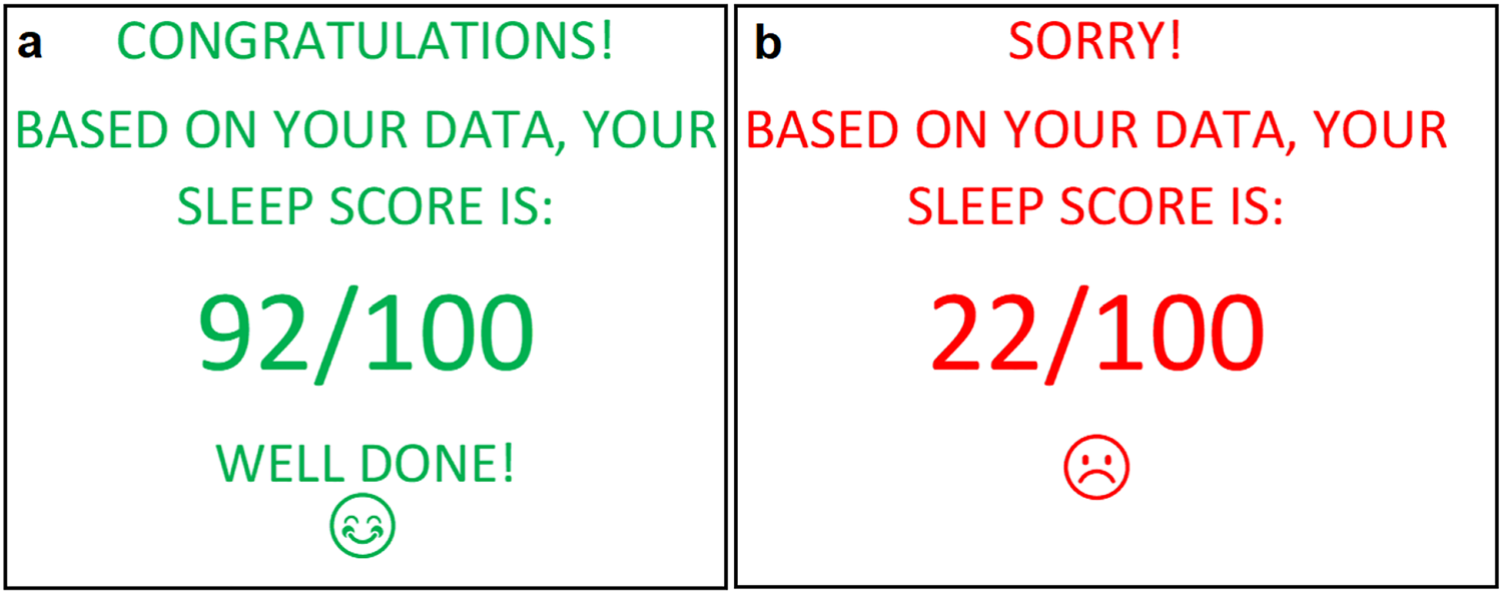
Good and poor false sleep feedback

All participants were initially told in the participant information sheet that the true purpose of the study was to assist in the development of an automatic sleep scoring algorithm, which would use their completed sleep diary data to generate an accurate, personalised, sleep score. Participants in the good sleep feedback condition were shown a message, in green text, stating “*Congratulations! Based on your data, your sleep score is 92/100. Well done!*” alongside an icon of a smiling face (Fig. 2a). Participants in the poor sleep feedback condition were shown a message, in red text, stating “*Sorry! Based on your data, your sleep score is: 22/100*”, alongside an icon of an unhappy face (Fig. 2b).

### Statistical analyses

To examine if poor sleep feedback resulted in higher levels of pre-sleep cognitive arousal, PSAS somatic and cognitive subscores were compared between good and poor feedback groups using a 2 (group) × 3 (time point: Night 0, Night 7, Night 14) mixed analysis of variance (ANOVA).

To examine if poor sleep feedback disrupted sleep continuity, averaged CSD-M sleep continuity values (TST, TIB, SE%, SOL, NWAK and WASO) were compared before and after the presentation of the sleep feedback (average days 1–7 compared to average days 8–14). Sleep diary data were analysed if participants included a minimum of five days of CSD-M data from days 1–7 and days 8–14. There was no significant difference in CSD-M missing data between good and poor feedback groups (*p* > 0.05). CSD-M analyses were conducted using 2 (group) × 2 (time) mixed ANOVAs, where *p* values were adjusted for multiple comparisons (adjusted *p* value = 0.008). Additionally, to examine the impact of sleep feedback n state anxiety, STAI-SF scores were compared between groups using a 2 (group) × 2 (time) mixed ANOVA.

## Results

Complete PSAS data were obtained from 46 participants (*M*_age_ = 30.96 years; SD_age_ = 13.43 years), consisting of 21 participants in the poor sleep feedback group (*M*_age_ = 30.35 years; SD_age_ = 13.89 years; 6 male/13 female/2 other) and 25 participants in the good sleep feedback group (*M*_age_ = 31.47 years; SD_age_ = 13.28 years; 7 male/17 female/1 other). Complete sleep diary data were obtained from 46 participants (*M*_age_ = 30.74 years; SD_age_ = 13.37 years), consisting of 21 participants in the poor sleep feedback group (*M*_age_ = 30.27 years; SD_age_ = 13.95 years; 6 male/13 female/2 other) and 25 participants in the good sleep feedback group (*M*_age_ = 31.47 years; SD_age_ = 13.29 years; 7 male/17 female/1 other). Demographic and relevant questionnaire results are shown in Table 1.

**Table 1:** Participant demographics (*n* = 46)

	Mean	SD
Age	30.96	13.42
Gender (male/female/other; *n*/%)	13 (28.3%)/30 (65.2%)/3 (6.5%)	
PSQI	9.28	2.81
GAD-7	7.46	5.15
PHQ-9	7.63	5.00

*PSQI* Pittsburgh sleep quality index; *GAD-7* generalised anxiety disorder 7-item; *PHQ-9* patient health questionnaire 9-item

There was no difference between good and poor sleep feedback groups in terms of pre-sleep cognitive arousal, as the interaction for PSAS cognitive scores between time point and group was not significant (*F*(2,88) = 0.34, *p* > 0.05; *η*^2^_p_ = 01), and the interaction for PSAS somatic subscores between time point and group was not significant (*F*(2,88) = 1.32, *p* > 0.05; *η*^2^_p_ = 0.03). The main effect of time point, and group, was not significant for PSAS somatic or cognitive subscores (Table 2). There was no significant interaction between group and time point on any subjective sleep continuity measure, or STAI-SF scores (all *p* values > 0.008; Table 3).

**Table 2:** Pre-sleep arousal at baseline, before and after sleep feedback

	Good sleep feedback (*n* = 25)						Poor sleep feedback (*n* = 21)						
	Night 0		Night 7		Night 14		Night 0		Night 7		Night 14		
	Mean	SD	Mean	SD	Mean	SD	Mean	SD	Mean	SD	Mean	SD	*p* value (interaction)
PSAS Cognitive	18.36	7.15	17.56	6.72	17.64	7.09	18.76	7.60	19.33	7.32	18.43	7.09	0.71
PSAS Somatic	11.08	3.69	11.40	4.36	12.08	4.73	12.81	5.47	11.95	5.83	12.19	5.43	0.27

*PSAS* pre-sleep arousal scale

**Table 3:** Subjective sleep continuity and state anxiety before and after sleep feedback

	Good sleep feedback (*n* = 25)				Poor sleep feedback (*n* = 21)				
	Week 1		Week 2		Week 1		Week 2		
	Mean	SD	Mean	SD	Mean	SD	Mean	SD	*p* value (interaction)
TIB (mins)	599.03	107.24	591.29	105.80	615.79	96.13	606.65	73.22	0.92
TST (mins)	460.06	34.14	472.78	44.12	454.96	55.42	462.83	65.08	0.68
SOL (mins)	27.74	24.86	20.90	17.05	28.01	20.64	24.40	21.98	0.61
NWAK	1.43	0.86	1.10	0.97	1.65	1.14	1.43	0.93	0.53
WASO (mins)	10.68	13.16	8.50	8.13	24.65	35.79	12.19	12.39	0.18
SE (%)	78.53	9.43	81.68	10.55	75.20	10.29	77.36	12.55	0.64
STAI-SF	10.20	0.97	10.43	1.12	10.53	0.93	10.30	0.97	0.05

*TIB* time in bed; *TST* total sleep time; *SOL* sleep onset latency; *NWAK* number of awakenings; *WASO* wake after sleep onset; *SE* sleep efficiency; *STAI-SF* state/trait anxiety (short-form)

## Discussion

The aim of the current pilot study was to examine if the presentation of poor sleep feedback, in the form of a score presented to participants as being directly calculated from their habitual sleep diaries, would negatively affect pre-sleep cognitive arousal and subjective sleep continuity in healthy sleepers. Contrary to expectations, poor sleep feedback did not affect pre-sleep cognitive arousal, any sleep continuity variable (including TST or SE%), or state anxiety, relative to good sleep feedback.

These results indicate that a single presentation of poor sleep feedback is unlikely to negatively affect pre-sleep cognitive arousal, or subjective sleep continuity, in healthy sleepers. However, to our knowledge, this is the first study to specifically examine whether poor sleep feedback, analogous to device-calculated sleep metrics [3], could specifically disrupt subjective sleep in healthy sleepers.

The main implication of the current study is that although sleep diary-derived sleep feedback should be considered a salient sleep-related cue, a single presentation of poor sleep feedback is unlikely to directly disrupt sleep or increase pre-sleep cognitive arousal. This finding is in contrast to previous studies that have found that monitoring sleep-related cues, or continuously monitoring digital device-derived metrics, can negatively affect subjective sleep and pre-sleep cognitive activity [7–10]. Speculatively, this would indicate that monitoring poor sleep feedback on an occasional basis is unlikely to increase pre-sleep cognitive arousal or negatively affect subjective sleep, in healthy sleepers.

There are a number of reasons for the unexpected findings observed in the present study. Firstly, it is possible that in healthy sleepers, a single presentation of negative sleep feedback may not be sufficient to disturb sleep. To disturb sleep, it is possible that personalised sleep feedback may need to be repeatedly displayed to participants, as this is potentially more representative of the manner in which the users of digital wearable devices are instructed to use them [3]; similarly, future studies should examine pre-sleep cognitive arousal on a nightly basis. Speculatively, this may suggest that healthy sleepers have a protective ‘buffer’ against negative sleep-related stimuli if these stimuli are not considered to be salient or personally relevant.

Secondly, individuals who had the specific intention to improve their sleep based on their feedback were not recruited. It is possible that these individuals would have been more negatively affected by the poor sleep feedback if they believed that there was a direct link between their sleep feedback and their own subjective experience of sleep [11], or if they were motivated to use this feedback with the specific intention of enhancing their own sleep [2].

There are four main ways in which this study could be extended in future. First, the ecological validity of the study could be increased by using actigraphy, since individuals may be more likely to believe the accuracy of the fictional sleep metric. This would also have the advantage of allowing for the measurement of objective sleep alongside subjective sleep; as to date, no studies have examined this in healthy sleepers. This would also allow for any potential discrepancy between the subjective experience of sleep, and the objective sleep that was attained, to be examined. Secondly, as stated, sleep feedback may need to be displayed more regularly to induce an effect in healthy sleepers. Thirdly, polysomnography (PSG) could be used to examine the impact of sleep feedback on objective sleep continuity. This is relevant as high levels of pre-sleep cognitive arousal have been shown to be associated with negative changes to PSG-measured objective SL, SE% and TST [9], and it is feasible to assess the impact of sleep feedback within a sleep laboratory environment [12]. Finally, there may also be individual factors which mean that the sleep of certain users will be more negatively affected by poor sleep feedback than other individuals. For example, individuals who report clinical sleep disturbances typically display personality aspects such as perfectionism or neuroticism [20, 21]. Indeed, recent evidence demonstrates that individuals who are preoccupied with improving, or perfecting, their sleep on the basis of sleep feedback, can develop sleep problems that require treatment [11]. However, to date, no studies have examined these individual factors to examine whether the sleep of certain healthy sleepers may be more affected than others by poor sleep feedback, in this context.

Strengths of the present study include the experimental design, where the true purpose of the study was hidden from participants, which enabled the specific effect of the sleep feedback to be assessed. Additionally, the study had a good level of ecological validity by monitoring the sleep of participants in the real world, and not in a laboratory setting. Limitations of the present study include the fact that we could not assess how often, and how long, participants viewed their sleep feedback for. We were also unable to assess the impact of the sleep feedback upon objective sleep in the current study, and future work should utilise both actigraphy and polysomnography to examine this in more detail.

## Conclusions

Overall, these results indicate that poor sleep feedback does not negatively affect pre-sleep cognitive arousal or subjective sleep continuity in healthy sleepers, relative to good sleep feedback.
